# Research on environmental mutagenesis from young scientists – the open symposium of the Japanese Environmental Mutagen Society (JEMS) in 2017

**DOI:** 10.1186/s41021-017-0086-8

**Published:** 2017-11-01

**Authors:** Kenichi Masumura, Shuichi Masuda

**Affiliations:** 1 Division of Genetics and Mutagenesis, National Institute of Health Sciences, 1-18-1 Kamiyoga, Setagaya-ku, Tokyo, 158-8501 Japan; 2 School of Food and Nutritional Sciences, University of Shizuoka, 52-1, Yada, Suruga-ku, Shizuoka, 422-8526 Japan

**Keywords:** DNA damage, Mutagenesis, Carcinogenesis, Environmental mutagen, Genetic toxicology

## Abstract

The open symposium of the Japanese Environmental Mutagen Society (JEMS) titled, “Research on Environmental Mutagenesis from Young Scientists,” was held at Kokusai Kenkyu Koryu Kaikan, the Foundation for Promotion of Cancer Research, in Tokyo on June 10, 2017. The aim of this symposium was to provide an opportunity to present the research activities of young scientists in the important field of environmental mutagenesis and genetic toxicology and inform JEMS activities to the participants. The organizers reported the symposium summary.

## Background

In genetic toxicology and environmental mutagenesis, novel analytical methods and tools are driving current advances in scientific research. Examples include sensitive biomarkers of DNA damage and response, quantitative analysis using mass spectrometry, multi-endpoint studies, animal models, and environmental monitoring. Updated reports from laboratories will provide new insights into the safety of chemicals, human health, and the environment. The open symposium of the Japanese Environmental Mutagen Society (JEMS) is held annually to present JEMS activities to researchers and the public [1–3]. In 2017, the symposium titled, “Research on Environmental Mutagenesis from Young Scientists” was held at Kokusai Kenkyu Koryu Kaikan, the Foundation for Promotion of Cancer Research, in Tokyo on June 10. The aim of the symposium this year was to provide an opportunity to present research activities of young scientists in the notable field of environmental mutagenesis and genetic toxicology and inform a wide range of people on JEMS activities. Nine young scientists from among JEMS members presented at the symposium. The organizers, Kenichi Masumura and Shuichi Masuda, reported the symposium summary.

## Symposium program

Uno Yoshifumi (President, JEMS: Mitsubishi Tanabe Pharma Corporation), Opening Speech.

Kenichi Masumura (National Institute of Health Sciences), Introduction.

Session 1 (Chairs: Shigeharu Muto and Manabu Yasui)

- Shun Matsuda (Fujifilm Corporation), Visualization and quantification of DNA damage-response signals for evaluation of genotoxicity.
- Yoshinori Okamoto (Meijo University), Development of non-genotoxic tamoxifen analogs based on mechanisms of DNA adduct formation.
- Yuji Ishii (National Institute of Health Sciences), Understanding early events in chemical carcinogenesis
- Naoki Koyama (Eisai Co., Ltd.), Study of colon carcinogenesis induced by non-mutagenic carcinogens using mouse inflammation model.

Session 2 (Chairs: Hiroyuki Kamiya and Shuichi Masuda)

- Tatsushi Toyooka (National Institute of Occupational Safety and Health), Phosphorylated histone H2AX induced by chemicals and its application in genotoxicology.
- Megumi Sasatani (Hiroshima University), Research on mechanisms for chemical- and radiation-induced carcinogenesis using highly susceptible mouse model
- Hiroko Ishiniwa (Fukushima University), Environmental chemical pollution and ecology of wild mice in forest.

Session 3 (Chairs: Takeshi Morita and Kenichi Masumura)

- Shigeki Motoyama (Chugai Pharmaceutical Co., Ltd.), Evaluation of DNA damages using γH2AX in stages of drug development.
- Hiroshi Honda (Kao Corporation), Environmental mutagenesis and genomics research driven by big data and algorithms.

Shuichi Masuda (University of Shizuoka), Closing Speech.

## Meeting report

Dr. Shun Matsuda reported his research on the visualization of DNA damage-response signals in cultured cells. This technology can be applied to simple and quick detection of genotoxicity. For example, DNA double-strand breaks orderly recruit several proteins and their modifications, such as phosphorylated histone H2AX, MDC1, and ATM, which coordinate DNA damage-response signals. He explained the absolute quantification of the components using mass spectrometry and how it could help to elucidate the stoichiometry and molecular mechanisms of DNA damage response.

Dr. Yoshinori Okamoto reported his research on the development of non-genotoxic tamoxifen analogs. Tamoxifen is a breast cancer drug, but one of its side effects is endometrial cancer. Based on the mechanisms of carcinogenicity with estrogenic activity and DNA adduct formations of the metabolite, new tamoxifen analogs were designed. These analogs exhibited higher anti-breast cancer potential and no DNA-adduct formation activity in animal studies, thereby making it a prime example of molecular design for non-genotoxic drugs.

Dr. Yuji Ishii presented his research on the early events involved in chemical carcinogenesis. DNA damage, such as DNA-adduct formation, which is an initial step leading to genotoxic carcinogenesis. He analyzed DNA adducts in genomic DNA extracted from tissues of mutagen-treated animals using LC-MS/MS. In addition, transgenic rodent gene mutation assays and histopathological analyses were used to monitor initiation and promotion processes in the target organ. He reported the cases of madder color, estragole, and acrylamide.

Dr. Naoki Koyama reported on a colon cancer model in mice. Oral administration of benzo[*a*]pyrene alone does not induce colon tumors in mice; however, when administered in combination with dextran sulfate sodium, colon tumors are induced within several weeks. This quick tumorigenesis model may be useful to investigate genetic and epigenetic mechanisms involved in colon carcinogenesis. This study suggested important roles of inflammation in the regulation of signaling pathways and epigenetic changes in colon cancer.

Dr. Tatsushi Toyooka reported that phosphorylated histone H2AX, γH2AX, is a noteworthy biomarker of DNA damage and can be applied to genetic toxicology studies. Histone H2AX is a variant of core histones participating in nucleosome formation. One DNA double-strand break can lead to the phosphorylation of thousands of H2AX molecules at serine 139, creating foci at the sites of double-strand breaks. The detection of such chemical modifications of proteins requires sensitive and high-throughput techniques, suggesting some advantages in the application of H2AX. It could be a useful tool for occupational safety scientists evaluating exposure to genotoxic chemicals.

Dr. Megumi Sasatani reported her research on the role of translesion DNA synthesis in the context of carcinogenesis. One of the error-prone DNA polymerases, Rev1, incorporates dCTP at the position of a DNA lesion in the template strand and plays an important role in lesion bypass. It is reported that the regulation of *Rev1* expression affects sensitivity to DNA damage and induction of mutations in vitro. Rev1-overexpressing transgenic mice were developed, and their sensitivity to chemical- and radiation-induced cancers was investigated to elucidate the contribution of *Rev1* in vivo.

Dr. Hiroko Ishiniwa presented research on the ecology of wild mice in forests and an application for environmental toxicology. Dioxins are highly toxic environmental organic pollutants and one of their sources is poorly operated waste incineration facilities. The aryl hydrocarbon receptor is included in the mechanism of toxicity, and a single amino acid variant shows a relatively resistant phenotype. Such genetic variants could contribute to the adaptation of wild animals to their environment. She investigated whether or not environmental pollution by dioxins affects the distribution of genetic variants in wild mice inhabiting an area where open burning of wastes was practiced.

Dr. Shigeki Motoyama presented his research on the utility of γH2AX as a marker of DNA damage in drug development stages. γH2AX is expected to be a highly sensitive and quantitative biomarker of DNA double-strand breaks. He presented an example of a positive compound from a micronucleus test, during the early screening stage, which can help to evaluate whether or not aneugenic actions are suggested using γH2AX analysis. He also reported on the in vivo study of DNA damage detected by immunohistopathological analyses of γH2AX.

Dr. Hiroshi Honda presented that big data science is very significant to environmental mutagenesis and genomics research. Optimization of algorithms could provide meaningful information from different types of omics data. He highlighted examples including the followings: (1) toxicogenomics, a correlation between gene expression profiles and liver hypertrophy and cancer; 2) genomics, genome-wide detection of mutations and specific mutational signatures; and 3) a structure–activity relationship (SAR) approach, a hybrid algorithm of statistical and knowledge-based models that predict genotoxicity.

There were approximately 100 participants at the symposium. A questionnaire survey revealed that 30% of the participants were not JEMS members. We would like to thank to everyone who attended this symposium.

## Abbreviations

JEMS: Japanese Environmental Mutagen Society
LC-MS/MS: Liquid chromatography - tandem mass spectrometry
SAR: Structure–activity relationship
γH2AX: Phosphorylated histone H2AX
